# Tissue Factor Expression in Obese Type 2 Diabetic Subjects and Its Regulation by Antidiabetic Agents

**DOI:** 10.1155/2015/291209

**Published:** 2015-03-15

**Authors:** Jing Wang, Theodore P. Ciaraldi, Fahumiya Samad

**Affiliations:** ^1^Torrey Pines Institute for Molecular Studies, San Diego, CA 92121, USA; ^2^San Diego Biomedical Research Institute, San Diego, CA 92121, USA; ^3^VA San Diego Healthcare System, San Diego, CA, USA; ^4^Department of Medicine, University of California, San Diego, La Jolla, CA 92093, USA

## Abstract

*Objective.* Increased coagulation activation may contribute to the high incidence of cardiovascular complications observed in obese and type 2 diabetes (T2D) subjects. Although tissue factor (TF), the primary initiator of coagulation is increased in obesity, its expression in adipose tissues and its association with metabolic parameters are unclear. We sought to compare TF expression in plasma and adipose tissues of obese subjects with and without T2D, its correlation with metabolic parameters, and regulation in response to antidiabetic drugs.* Methods* Subjects were recruited from diabetes clinics and adipose tissue was obtained by needle biopsy of lower subcutaneous abdominal depot. For the intervention study, subjects were randomized into treatment groups with rosiglitazone or metformin for 4 months.* Results.* Plasma TF antigen, activity, and adipose TF mRNA were greater in obese T2D subjects compared with obese nondiabetics. Plasma TF activity correlated with fasting insulin, glucose, and free fatty acids, (FFAs), and adipose TF mRNA correlated with plasma FFA. Plasma TF activity was reduced by metformin and increased with rosiglitazone treatment.* Conclusions.* Specific diabetes-related metabolic parameters, but not obesity per se, are correlated with TF expression. Regulation of TF activity by different classes of antidiabetic drugs may relate to protective or adverse cardiovascular outcomes.

## 1. Introduction

Obesity is a major risk factor for the development of type 2 diabetes (T2D), and clinical studies have established an increased incidence of thrombosis and cardiovascular disease as a primary cause of mortality in diabetic patients [1, 2]. T2D is associated with accelerated and premature atherosclerosis as well as other cardiovascular and thrombotic complications including myocardial infarction, ischemic stroke, and peripheral vascular disease [1, 2]. While risk factors such as hypertension and cholesterol are elevated in T2D, these only account partially for the cardiovascular burden, and emerging evidence suggests that dysregulation of pathways of coagulation is an additional significant mechanism that contributes to increased cardiovascular risk.

TF is the primary initiator of the coagulation pathway and is the cell surface receptor for coagulation factors VII and VIIa. Cell surface or microparticle- (MP-) associated TF-FVIIa complex triggers extrinsic coagulation leading to FXa-mediated generation of the downstream coagulation protease thrombin, fibrin deposition, and platelet activation. Higher plasma concentrations of FVII [3], increased levels of thrombin and thrombin-antithrombin (TAT) complexes [4], and increased circulating monocyte TF procoagulant activity [5] are observed in obese subjects. Weight loss in morbidly obese patients significantly reduces thrombin generation potential [6] and decreases levels of circulating TF, FVII, and prothrombin fragment F1.2, a marker of in vivo thrombin formation [7]. Compared with nondiabetic control subjects, T2D patients display signs of hypercoagulability and increased plasma TF procoagulant activity [8, 9], increased abundance of TF-positive microparticles [8, 10], and higher TF activity of circulating monocytes [11, 12]. Elevated blood-borne or circulating TF correlates with microvascular complications and is considered a biomarker for the severity of microvascular disease in T2D patients [13, 14].

Whereas plasma TF activity and antigen levels have been shown to be increased in obese subjects as well as in patients with T2D, whether its expression is higher in obese diabetics compared with obese nondiabetics is unclear. This is especially important since all obese subjects are not necessarily diabetic. In genetically or high fat diet induced obese mice that are also diabetic, TF activity is increased in the blood and its activity and gene expression increased in adipose tissues [15–17]. However, to our knowledge whether adipose TF expression is similarly induced in obese and diabetic humans has not been studied. Here, we examined TF expression in plasma and adipose tissues of obese subjects with and without diabetes, how its expression correlates with a number of metabolic parameters, and how its activity is regulated in response to the antidiabetic drugs, metformin, and rosiglitazone.

## 2. Patients and Methods

Human subject protocols were approved by the Committee on Human Investigation at the University of California, San Diego (UCSD), and all subjects provided written informed consent. Subjects were recruited from diabetes clinics and classified as diabetic or nondiabetic by their response to a 75 g oral glucose tolerance test according to the American Diabetes Association criteria and classified as obese if their BMI (kg/m^2^) > 30 as previously described [18]. Adipose tissue was obtained by needle biopsy of the lower subcutaneous abdominal depot [18]. Other clinical data are summarized in Table 1(a). The time from initial diabetes diagnosis to sample collection ranged from 0.2 to 13 years. None of the subjects had a history of thromboembolism. All routine laboratory test values were within 2.5X the upper and lower limits of specified normal ranges. A majority of the T2D subjects were not taking any antidiabetic medications at the time of biopsy and were controlled by diet alone as part of a 6-week washout of antidiabetic medications. Of the other T2D subjects, 1 was on glucovance and 1 was taking rosiglitazone and the other glyburide and metformin at the time of biopsy.

In the intervention study (Table 1(b)), subjects were excluded if they were previously treated with TZD, treated with more than one diabetic agent or insulin, had hypertension, were pregnant, or had active cardiac disease or other major illnesses. If applicable, subjects maintained their blood pressure and lipid-controlling medications. There were no differences at baseline between treatment groups with regard to duration of diabetes, blood pressure, routine laboratory values, or use of blood pressure or lipid-controlling medications. Briefly, after a washout period of 6 weeks for those participants that were on an antidiabetic treatment, subjects were randomized into a treatment group with high-dose rosiglitazone (4 mg twice daily) or high-dose metformin (1,000 mg twice daily) for a period of 4 months [19]. To minimize potential side effects, medications were initiated below target doses and titrated up over the initial 2 weeks. Both subjects and investigators were blinded to treatment. Blood was collected after 10–12-hour overnight fast at baseline and 4-month posttreatment. All metabolic parameters for subjects in Table 1 were determined using standard techniques as previously described [18, 19].

Plasma TF antigen was determined using the IMUBIND Tissue Factor ELISA kit, and TF procoagulant activity in plasma was determined using the chromogenic Actichrome TF activity assay according to manufacturer's instructions (American Diagnostica, Stamford, CT). Adipose TF gene expression was determined by real-time quantitative RT-PCR as previously described [17]. cDNA synthesized from total RNA was analyzed with gene specific primers (Invitrogen) and SYBR Green PCR Master mix (PerkinElmer) in an iCycler (Bio-Rad). Relative gene expression levels were calculated after normalizing to *β*-actin using the ΔΔCT method (Bio-Rad). Statistical analysis was evaluated using the GraphPad Prism program (Intuitive Software, San Diego, CA). The statistical significance of differences within and between groups was analyzed by the Student's paired and unpaired *t*-tests. Correlations were calculated using the Pearson correlation. A *P* value < 0.05 was considered as the level of significance.

## 3. Results and Discussion

**Figure 1:**
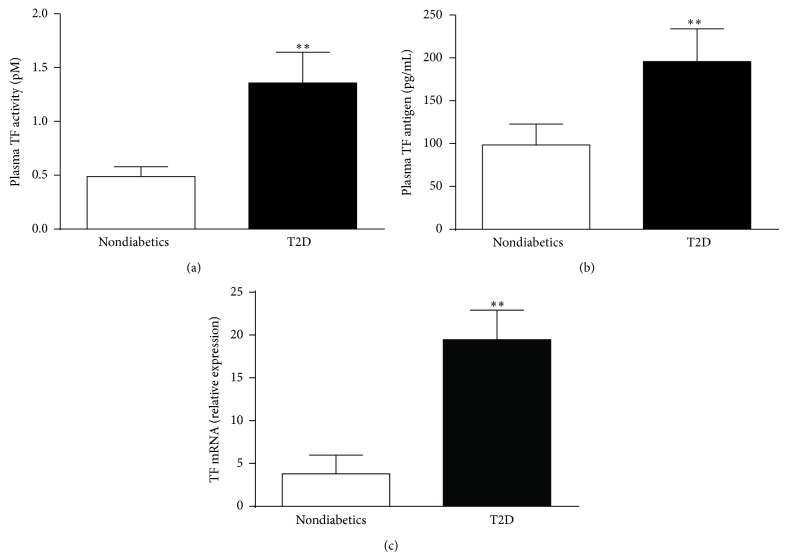
Plasma TF activity (a), antigen (b), and adipose TF mRNA levels (c) in obese T2D subjects compared with obese nondiabetics. For panels (a) and (b),* N* = 10–13 ± SD. For panel (c),* N* = 6–10 ± SD. ^**^ *P* < 0.01 nondiabetics versus T2D. Clinical characteristics of subjects are indicated in Table 1(a).

Although obesity poses a risk for the development of insulin resistance and T2D, not all obese subjects are diabetic. The influence of T2D on tissue factor expression in obese subjects was evaluated in cohorts matched for weight and BMI as indicated in Table 1(a). The body weights (kg) of obese nondiabetics and diabetics were 105 ± 17.3 and 101 ± 15.9, while their BMIs (kg/m^2^) were 35.6 ± 5.6 and 35.6 ± 7.1, respectively. Fasting levels of plasma glucose, insulin, free fatty acids (FFAs), and HbA_1c_ were significantly higher in the obese diabetic cohorts, while no differences were observed for plasma triglycerides between the two groups. Plasma TF activity, plasma TF antigen, and adipose TF mRNA expression were significantly increased in obese diabetics compared with obese nondiabetics (Figures 1(a)–1(c)).

In the commercial Actichrome TF activity assay (American Diagnostica) plasma samples are directly incubated with FVIIa, FX, and a chromogenic substrate for FXa. A limitation of this assay is that the values obtained tend to be somewhat overestimated. However, regardless of the absolute levels of TF activity, we believe that, on a relative basis, the observation that plasma TF activity is higher in obese T2D relative to obese nondiabetics is relevant. In the plasma, TF exists in the form of microparticles shed from apoptotic or activated cells, as well as an alternately spliced soluble form of TF. It should be noted that the TF activity and antigen assays used did not discriminate between these various forms of TF but rather measured total plasma TF.

**Figure 2:**
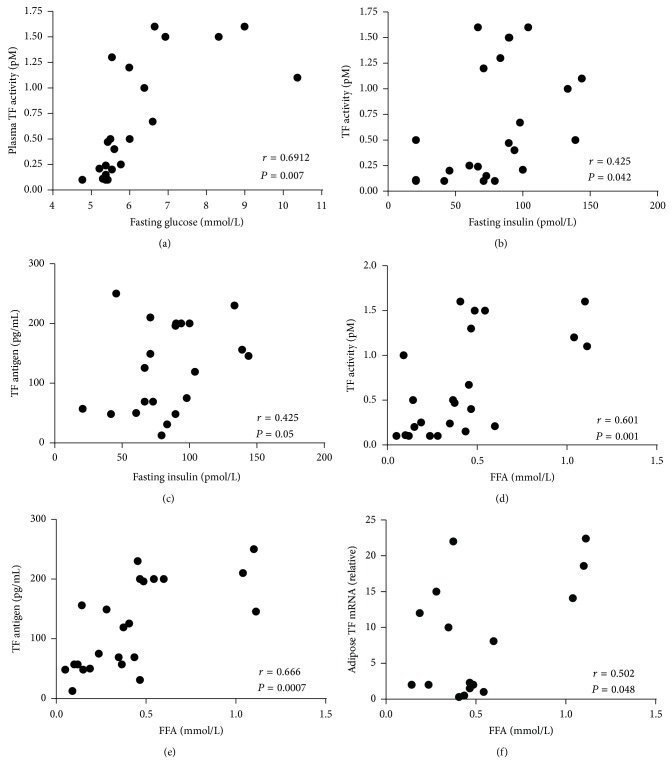
Correlations between (a) plasma TF activity levels and plasma fasting glucose, (b) plasma TF activity and fasting insulin, (c) plasma TF antigen and fasting insulin, (d) plasma TF activity and plasma free fatty acid (FFA), (e) plasma TF antigen and plasma FFA, and (f) adipose TF mRNA and plasma FFA. Correlations were calculated using the Pearson correlation. A *P* value < 0.05 was considered as the level of significance.

Since metabolic changes observed in T2D may contribute to elevated TF expression, we determined the correlation of TF expression with a number of metabolic parameters. As indicated in Table 2, no significant correlations were observed for plasma TF activity and antigen with either BMI or weight suggesting that obesity per se may not contribute to elevated TF activity. Alternately, TF activity was significantly correlated with fasting glucose, insulin, and free fatty acid (FFA), while TF antigen was correlated with fasting insulin and FFA (Table 2, Figure 2). Interestingly adipose TF mRNA was correlated only with plasma FFA (Table 2, Figure 2(f)).

Hyperinsulinemia is associated with insulin resistance and T2D, and insulin administration has been demonstrated to increase circulating TF in healthy and T2D subjects [9, 20] and in obese insulin-resistant and diabetic mice, insulin increases both circulating and adipose TF expression [15, 16]. Microparticles shed from activated monocytes are important sources of blood-borne TF. Compared with normal monocytes, insulin-resistant T2D monocytes express more TF and shed MP with a higher TF levels in response to insulin [11]. TF expression is negatively regulated by phosphoinositide 3-kinase signaling [21], suggesting that downregulation of this pathway as a consequence of insulin resistance may favor TF upregulation in T2D [11]. Our study indicating that plasma TF activity and antigen are correlated with insulin levels is consistent with these previous data and suggests that the compensatory hyperinsulinemia that accompanies insulin resistance and T2D may contribute to the observed increase in plasma TF expression in these patients. A significant correlation was also observed between TF activity and glucose, and this is in line with data indicating that hyperglycemia in healthy volunteers and T2D increases TF procoagulant activity [9] and improved glycemic control reduces circulating TF [22]. Importantly, combined hyperinsulinemia and hyperglycemia produced an additive increase in TF activity [20], suggesting that the presence of these two conditions that often occur concurrently in T2D may contribute to the observed increase in TF and procoagulant activity in these patients.

In this study, the metabolic parameter that showed the strongest correlation with plasma TF activity and antigen and the only correlate associated with adipose TF expression was FFA (Table 2, Figure 2(f)). Obesity is associated with a dyslipidemia that in part is manifested as increased circulating FFA. Increased adipose lipolysis due to insulin resistance is a primary mechanism leading to elevated FFA in the plasma and in the local microenvironment of obese T2D patients. FFA activates toll-like receptor- (TLR-) mediated signaling, and in monocytes induction of TF expression has been shown to be dependent on the coordinated activation of TLR-dependent nuclear factor-*κ*B (NF-*κ*B) and Jun N-terminal kinase (JNK) signaling pathways [21]. It is tempting to speculate that FFAs could similarly increase TF expression in adipocytes and adipose tissue macrophages via TLR-mediated activation of NF-*κ*B and JNK signaling cascades. A role for FFA in TF induction was confirmed by our finding that TF expression in adipocytes is increased by palmitate (Samad, unpublished observation), the most abundant plasma FFA in obesity.

**Figure 3:**
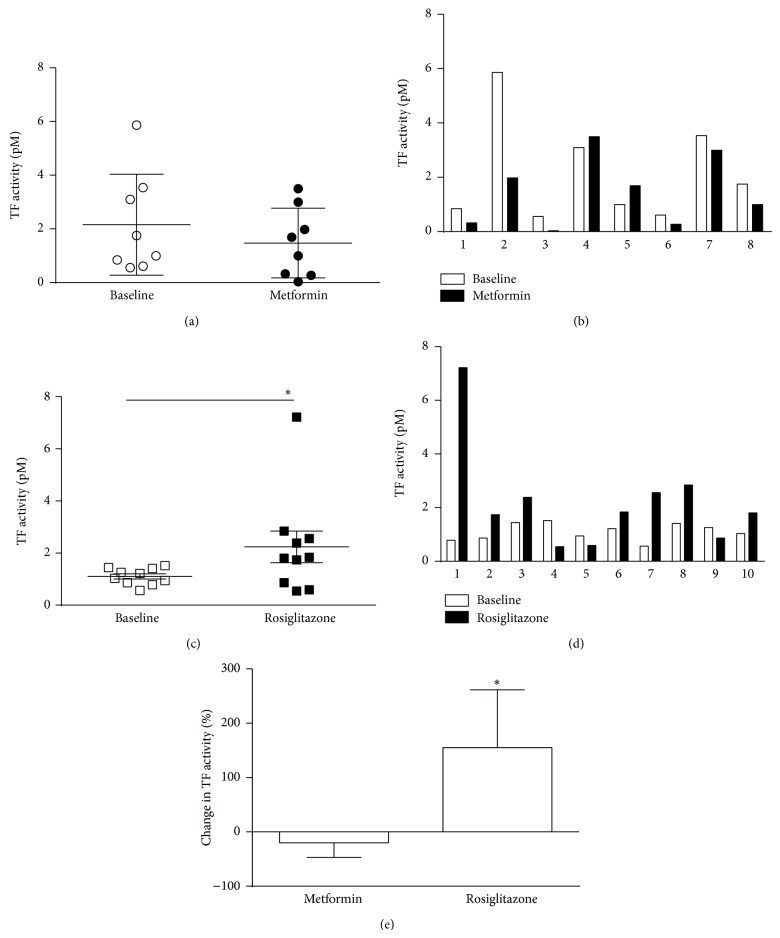
Plasma TF activity at baseline and following treatment with metformin ((a), (b), and (e)) and rosiglitazone ((c), (d), (e)). The *x*-axis numbers (1–8) in Figure 3(b) and (1–10) in Figure 3(d) represent each patient and are not identical as these are subjects from two treatments studies. For metformin-treated subjects in panels (a), (b), and (e):* N* = 8 ± SD. For rosiglitazone-treated subjects in panels (c), (d), and (e):* N* = 10 ± SD. ^*^ *P* < 0.05, baseline versus rosiglitazone. Baseline clinical characteristics of subjects are indicated in Table 1(b).

Metformin, a member of the biguanide family, is one of the most commonly used antidiabetic drugs and is highly effective in lowering blood glucose and improving peripheral insulin sensitivity in patients with T2D [23]. In addition, increasing studies indicate that metformin reduces the risk of diabetes-associated atherothrombotic disease independent of its antihyperglycemic effect [24]. A limited number of in vitro and clinical studies have suggested that the cardioprotective effects of metformin may be related to beneficial effects on pathways of fibrinolysis and hemostasis. In vitro studies show that metformin suppresses TF expression in human monocytes and reduces thrombin activity and fibrin polymerization [25, 26]. Modest reductions of VIIa activity were observed in metformin-treated T2D patients and in prediabetics on statin and metformin therapy [27]. To directly determine in vivo efficacy of metformin on suppressing coagulation, we determined the effect of metformin on TF activity in obese T2D subjects. Baseline characteristics of the treatment group are summarized in Table 1(b). Four-month high-dose metformin treatment led to a moderate insignificant reduction of plasma TF activity (Figures 3(a) and 3(e)). Out of eight patients that were followed, six showed a reduction in TF activity, while in two of the subjects TF activity was modestly increased (Figure 3(b)). These small decreases in TF activity observed in 75% of the patients however may be clinically relevant as such changes in hemostatic factors including factor VIIa have been shown to determine cardiovascular risk [28].

Thiazolidinediones (TZDs) that act through the nuclear receptor peroxisome proliferator-activated receptor-*γ* (PPAR*γ*) are potent insulin sensitizers and highly effective oral medications for T2D [29]. However, clinical trials have indicated that the TZD and rosiglitazone (Avandia) lead to a high risk of myocardial infarction and cardiovascular disease leading to their withdrawal [29]. While the reason for its adverse cardiovascular side effects is not fully understood, in vitro studies have demonstrated that rosiglitazone upregulated the expression of procoagulant TF bearing MP by human monocytes/macrophages [30]. To determine if rosiglitazone regulates TF activity in vivo, we measured plasma TF activity in a cohort of obese T2D subjects at baseline and after 4 months of rosiglitazone treatment. A significant increase in TF activity was observed after rosiglitazone treatment compared with baseline levels (Figures 3(c) and 3(e)). Of the 10 patients that were studied, 7 of them showed an increase and 3 showed a decrease in plasma TF activity (Figure 3(d)). To our knowledge, this is the first in vivo demonstration where rosiglitazone induces TF activity in human subjects which may potentially contribute to the adverse cardiovascular outcomes with this drug. The mechanism for rosiglitazone-mediated increase in TF activity is unclear and is not related to metabolic measures of insulin sensitivity as these were markedly improved in rosiglitazone-treated patients [19]. Despite their side effects, TZDs are still considered to have therapeutic value due to their superior antidiabetic effects. Since increased TF activity was not observed in all rosiglitazone-treated subjects, it is tempting to speculate that measures of TF activity could be a useful indicator to identify patients that may be at risk for developing cardiovascular disease in response to TZDs.

In conclusion, our study shows that plasma and adipose TF expression is increased in obese T2D compared to obese nondiabetic subjects and significantly correlated with FFA. Regulation of TF activity by different classes of antidiabetic drugs may relate to protective or adverse cardiovascular outcomes.

## Figures and Tables

**(a):** 

	ND	T2D
*n* (F/M)	10 (2/8)	13 (5/8)
Age (years)	45 ± 10.3	57 ± 9.5
BMI (kg/m^2^)	35.6 ± 5.6	35.6 ± 7.1
Weight (kg)	105 ± 17.3	101 ± 15.9
Fasting glucose (mmol/L)	5.5 ± 0.7	7.7 ± 1.6^*^
Fasting insulin (pmol/L)	84 ± 23.0	148 ± 31^*^
FFA (mmol/L)	0.32 ± 0.18	0.66 ± 0.25^*^
HbA_1c_ (%)	5.5 ± 0.6	7.2 ± 1.3^*^
TG (mg/dl)	132 ± 77	191 ± 90

**(b):** 

	Metformin (*n* = 8)	Rosiglitazone (*n* = 10)
BMI (kg/m^2^)	35.45 ± 5.4	32.91 ± 6.3
Weight (Kg)	103 ± 14.9	97.5 ± 18.9
Fasting glucose (mmol/L)	7.4 ± 2.2	9.3 ± 2.4
Fasting insulin (pmol/L)	140.6 ± 70.1	95.1 ± 39.5
FFA (mmol/L)	0.484 ± 0.12	0.482 ± 0.142
HbA_1c_ (%)	6.48 ± 0.65	8.0 ± 1.2
TG (mg/dl)	158 ± 49.6	142 ± 71

For Tables 1(a) and 1(b), data are means ± SD. TG, triglyceride.

Table 1(a): ^*^ *P* < 0.05; ND versus T2D.

**Table 2:** Correlation between TF and metabolic parameters.

	TF activity	TF antigen	Adipose TF mRNA
	*P* value	*P* value	*P* value
*n*	23	23	16
BMI	0.558	0.801	0.1
Weight	0.602	0.760	0.059
Fasting glucose	**0.007** ^**^	0.244	0.677
Fasting insulin (pmol/L)	**0.042** ^*^	**0.050**	0.888
FFA (mmol/L)	**0.001** ^**^	**0.0007** ^***^	**0.048** ^*^
HbA_1c_ (%)	0.483	0.758	0.172
TG (mg/dl)	0.746	0.251	0.604

^*^ *P* < 0.05, ^**^ *P* < 0.01, ^***^ *P* < 0.001 for TF (activity, antigen or adipose mRNA) versus indicated metabolic parameters.
